# Approaches and Recent Developments for the Commercial Production of Semi-synthetic Artemisinin

**DOI:** 10.3389/fpls.2018.00087

**Published:** 2018-01-31

**Authors:** Stephanie H. Kung, Sean Lund, Abhishek Murarka, Derek McPhee, Chris J. Paddon

**Affiliations:** Amyris Inc., Emeryville, CA, United States

**Keywords:** artemisinic acid, artemisinin, semi-synthetic, *Saccharomyces cerevisiae*, synthetic biology, *Artemisia annua*

## Abstract

The antimalarial drug artemisinin is a natural product produced by the plant *Artemisia annua*. Extracts of *A. annua* have been used in Chinese herbal medicine for over two millennia. Following the re-discovery of *A. annua* extract as an effective antimalarial, and the isolation and structural elucidation of artemisinin as the active agent, it was recommended as the first-line treatment for uncomplicated malaria in combination with another effective antimalarial drug (Artemisinin Combination Therapy) by the World Health Organization (WHO) in 2002. Following the WHO recommendation, the availability and price of artemisinin fluctuated greatly, ranging from supply shortfalls in some years to oversupply in others. To alleviate these supply and price issues, a second source of artemisinin was sought, resulting in an effort to produce artemisinic acid, a late-stage chemical precursor of artemisinin, by yeast fermentation, followed by chemical conversion to artemisinin (i.e., semi-synthesis). Engineering to enable production of artemisinic acid in yeast relied on the discovery of *A. annua* genes encoding artemisinic acid biosynthetic enzymes, and synthetic biology to engineer yeast metabolism. The progress of this effort, which resulted in semi-synthetic artemisinin entering commercial production in 2013, is reviewed with an emphasis on recent publications and opportunities for further development. Aspects of both the biology of artemisinin production in *A. annua*, and yeast strain engineering are discussed, as are recent developments in the chemical conversion of artemisinic acid to artemisinin.

## Introduction

**FIGURE 1:**
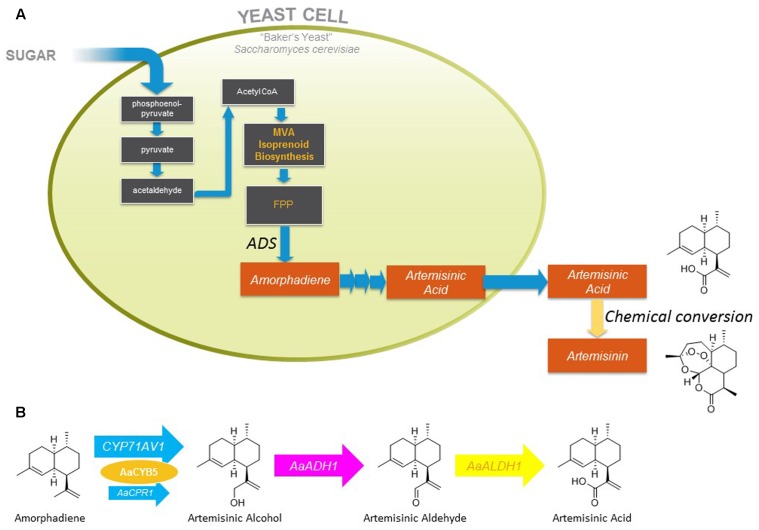
Summary of yeast strain engineering underlying fermentative production of artemisinic acid and subsequent conversion to artemisinin. **(A)** Biosynthetic pathway engineered in yeast for production of amorphadiene, its oxidation to artemisinic acid, and subsequent chemical conversion to artemisinin. **(B)** *A. annua* enzymes expressed in yeast for oxidation of amorphadiene to artemisinic acid. MVA, mevalonate; FPP, farnesyl diphosphate; *Aa*ADH1, alcohol dehydrogenase; *Aa*ALDH1, aldehyde dehydrogenase.

*Artemisia annua* has been known to traditional Chinese medicine for two millennia, but its modern history dates back to the 1970s when Chinese scientists rediscovered its antimalarial properties, and shortly thereafter isolated artemisinin, the active compound, and elucidated its structure (Tu, 2016, 2017). In 2002 the World Health Organization designated Artemisinin Combination Therapy (ACTs) as the first-line treatment for uncomplicated malaria (Paddon and Keasling, 2014). Following this decision there were significant swings in both the availability and price of artemisinin, which led to the concept of developing a second, non-plant derived, source to stabilize the availability and cost, and ultimately to decrease its cost (Hale et al., 2007). The semi-synthetic artemisinin project that sprang from this concept envisaged production of a late-stage precursor (artemisinic acid) by microbial fermentation, followed by its isolation from the fermentation medium and chemical conversion to artemisinin (**Figure 1A**; Hale et al., 2007).

The semi-synthetic artemisinin project that led to the industrial production of artemisinic acid and its chemical conversion to artemisinin has been reviewed (Paddon and Keasling, 2014). Briefly, brewer’s yeast (*Saccharomyces cerevisiae*) was engineered to overexpress the enzymes of the mevalonate pathway along with *A. annua* amorphadiene synthase, leading to the production of over 40 g/L amorphadiene (the hydrocarbon precursor of artemisinic acid) in fed-batch fermentors fed with ethanol (presumed to feed directly into cytosolic acetyl-CoA production) (Westfall et al., 2012). The cytochrome P450 enzyme (CYP71AV1) and its cognate reductase (*Aa*CPR) responsible for the oxidation of amorphadiene had been identified earlier (Ro et al., 2006), but conversion of amorphadiene to artemisinic acid was poor when expressed in yeast (Westfall et al., 2012). High-level production of artemisinic acid (25 g/L) by yeast fermentation at 2 L scale was achieved by decreasing the expression of *Aa*CPR to (presumably) alter the stoichiometry of the CYP71AV1:*Aa*CPR interaction, and co-expression of other enzymes (cytochrome b5 and two dehydrogenases) involved in the three oxidation reactions that convert amorphadiene to artemisinic acid (**Figure 1B**; Paddon et al., 2013). Following the development of an industrial process for the chemical conversion of artemisinic acid to artemisinin, commercial production of semi-synthetic artemisinin began in 2013 (Paddon and Keasling, 2014; Peplow, 2016).

The strain engineering and processes for production for amorphadiene and artemisinic acid at laboratory scale (Westfall et al., 2012; Paddon et al., 2013) were completed in 2008, almost a decade ago. Much has changed technologically in the intervening years, allowing the prospect of significantly improving the production of semi-synthetic artemisinin to decrease its cost. Developments have been made in several relevant areas including improved production of terpenes by yeast, understanding of cytochrome P450 oxidation reactions, advancements in understanding of the enzymology and physiology of artemisinin production in *A. annua* trichomes (Czechowski et al., 2016), and finally advances in the chemistry for conversion of artemisinic acid to artemisinin. These developments are described below. For comparison, advances in the biotechnological production of artemisinin in plants have been recently reviewed (Ikram and Simonsen, 2017), as has an overview of the engineering of cellular metabolism (Nielsen and Keasling, 2016).

## Developing Terpenoid Production Directly From Sugar

Early descriptions of amorphadiene production by yeast were at concentrations of ∼100 mg/L (Ro et al., 2006), which was increased to 40 g/L following considerable strain engineering and the use of a pure ethanol feedstock in pulse-fed batch fermentations. However, this methodology is not industrially scalable owing to the excessively high oxygen demand, and the process difficulties that a pure ethanol feed would bring (Westfall et al., 2012). An ethanol–glucose feed could be industrially feasible, but decreases amorphadiene production to less than 20 g/L (Westfall et al., 2012). While strains were generated that produce amorphadiene constitutively in lab-scale fermentations, it is likely that the strain stability necessary in a large-scale industrial fermentation would require the use of a switch to turn on production. The most well-studied genetic switches rely on the addition of galactose to the fermentation medium, which is expensive and would add to the production cost. There has been considerable progress in the industrial production of terpenes by yeast in the decade following the amorphadiene work described above, though directed primarily at production of another sesquiterpene, β-farnesene (Benjamin et al., 2016; Leavell et al., 2016).

Regarding the industrial process of artemisinic aid production, the use of sugar as the primary feedstock (as opposed to a glucose/ethanol mix) would lead to feedstock cost saving, albeit somewhat limited. Of greater significance is that recently developed strains using sugar as feedstock have much higher flux to product, attaining over 100 g/L β-farnesene production in 6-day fed-batch fermentations (Meadows et al., 2016). Conversion of these improved β-farnesene strains to amorphadiene production by swapping β-farnesene synthase for amorphadiene synthase should enable production of much greater concentrations of amorphadiene as a substrate for biological oxidation in an industrially scalable manner. The use of a cost-effective switch (Sandoval et al., 2014) to turn on production at scale would likely improve genetic stability, extend the fermentation production run, and decrease cost. Another process approach to improve production of artemisinic acid could involve *in situ* product removal using oils such as isopropyl myristate which has been demonstrated to boost artemisinic acid production (Paddon et al., 2013). Expression of an *A. annua* transport system for export of artemisinic acid (Wang et al., 2016) may improve its secretion from yeast.

A fundamental biochemical challenge is to improve the oxidation of amorphadiene to artemisinic acid. The highest published fermentative production of artemisinic acid in a yeast strain expressing the full complement of *A. annua* enzymes for the oxidation of amorphadiene was 25 g/L (Paddon et al., 2013), grown under the same regimen as the parental strain that produced 40 g/L amorphadiene (Westfall et al., 2012), a conversion efficiency of 55 mol%; there is clearly room for improvement in the oxidation of amorphadiene to artemisinic acid. Given that expression of *A. annua* alcohol and aldehyde dehydrogenases in yeast strains producing amorphadiene and expressing optimized CYP71AV1/*Aa*CPR/cytochrome-*b_5_* ratios virtually eliminated buildup of artemisinic alcohol and aldehyde (Teoh et al., 2009; Paddon et al., 2013), it seems reasonable to conclude that the bottleneck in oxidation of amorphadiene lies in the activity of CYP71AV1 and associated proteins. It follows that improving oxidation of amorphadiene to artemisinic alcohol by CYP71AV1 and associated proteins would likely be a fruitful approach to improving the overall production of artemisinic acid.

## Increasing Activity of Heterologously Expressed Cyp71Av1

At the current level of production, the concentration of amorphadiene is approaching 200 mM in the fermentation tanks, which is well beyond the solubility limit. At such high concentrations of substrate, plant P450s harvested from nature are working well outside of the biological context in which they evolved, in addition to being heterologously expressed in yeast. While CYP71AV1 is remarkably able to accomplish such high conversions under the extreme concentrations of amorphadiene, a variety of approaches to engineer improved P450 conversion and generate even higher, economically relevant titers of artemisinic acid are required.

Engineering of the catalytic system directly could be accomplished on several fronts. Based on previous successes with titrating expression levels of *Aa*CPR and cytochrome-*b_5_*, we know that modulation of the enzymes indirectly participating in catalysis can have a huge impact. While the interaction between CYP71AV1 and cytochrome-*b_5_* has not been characterized, our results (Paddon et al., 2013) are consistent with studies on the interaction between cytochrome-*b_5_* and CYP2B4, whereby cytochrome-*b_5_* provides the second electron of the oxidation reaction, the reaction being strongly influenced by the stoichiometry of the two proteins (Im and Waskell, 2011). Cytochrome-*b_5_* may also behave as an allosteric activator, as was suggested by its interaction with CYP3A4 (Zhao et al., 2012). *Aa*CPR has been shown to have relatively poor coupling to its cognate P450 compared to human CPRs and P450s (Simtchouk et al., 2013). This uncoupling likely produces large amounts of reactive oxygen species in addition to consuming valuable NADPH. Potential avenues to increase heterologous oxidation of amorphadiene would be to engineer the *Aa*CPR/CYP71AV1 coupling efficiency by altering the protein–protein interactions or weakening the binding of NADPH to *Aa*CPR when *Aa*CPR is not set up to transfer electrons to CYP71AV1. To reduce potential substrate or product inhibition that may be occurring under these abnormal conditions, engineering of CYP71AV1 directly through tightening of the active site or engineering of the substrate entrance channel could be undertaken. Such strategies would eliminate non-productive binding conformations observed in other P450s (Tietz et al., 2017). In addition to engineering the active site of P450s, engineering how P450s interact with the yeast endoplasmic reticulum has been fruitful for endeavors such as heterologous hydrocodone production (Galanie et al., 2015). Manipulation of the yeast genome may also be a means to improve heterologous P450 activity, for example it was recently shown that a mutation (Δpah1) resulting in expansion of yeast endoplasmic reticulum leads to an increase in the heterologous production of triterpenoid saponins (Arendt et al., 2017).

The approaches described above illustrate the need for development of rapid screening systems. Saturation mutagenesis of CYP71AV1, a protein of 496 amino acids, and testing production of oxidized product(s) from yeast producing amorphadiene with reasonable statistical coverage would require well over 10,000 assays to detect mutants with improved oxidation properties. Assays for quantification of artemisinic acid and other oxidized intermediates used to date are long [up to 30 min. (Paddon et al., 2013)], and too consuming of time and resources for high-throughput screening. A rapid methodology for detecting improved production of oxidized products of amorphadiene would be needed such as rapid mass spectrometry (Rohman and Wingfield, 2016) or surrogate assays based on spectrophotometric or fluorescent methods that could cut the time required to measure titers to 10 s or less per sample, albeit with the statistical reproducibility required to detect genuine improvements on artemisinic acid production from the background variability inherent in a high-throughput screen.

## Chemical Conversion of Artemisinic Acid to Artemisinin

**FIGURE 2:**
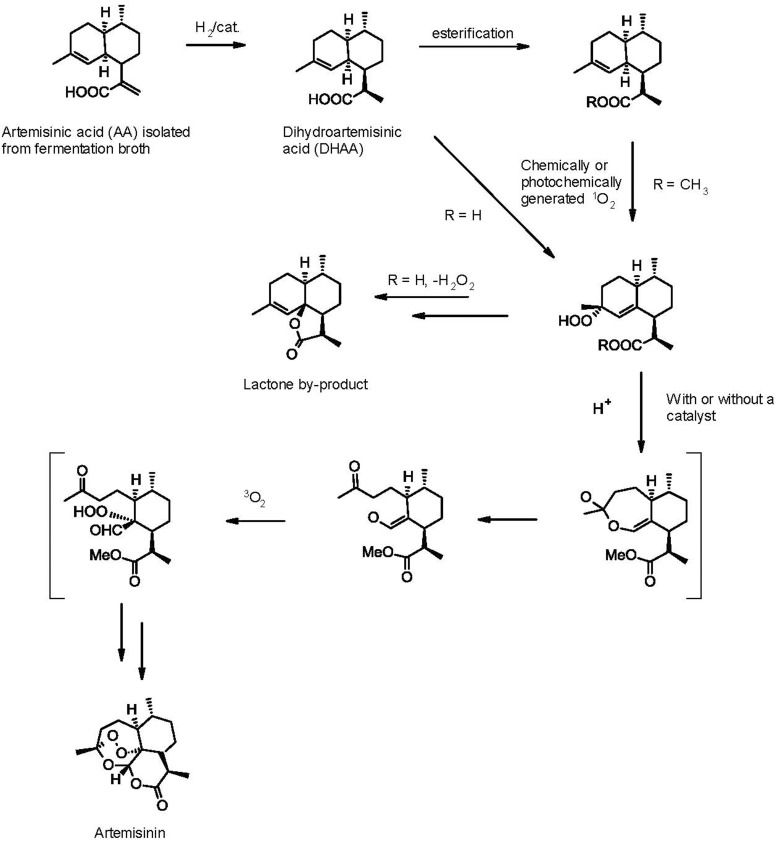
Generic chemical synthesis of artemisinin from artemisinic acid.

Following determination of the structure of artemisinin in 1977 (Tu, 2016, 2017), chemists quickly responded to the synthetic challenge presented by its sesquiterpene lactone structure, with seven chiral centers and a unique stable endoperoxide bond, and the first syntheses were reported soon thereafter. Broadly speaking, all these can be divided into two groups: total syntheses starting from a chiral pool compound and semi-syntheses from a terpene natural product precursor. The former is solely of academic interest, as they invariably involve too many steps to provide artemisinin at a price that can compete with extraction of the natural product from *A. annua* or the semi-synthetic approach described above. As there is no shortage of reviews that comprehensively cover both the earlier and more recent total and partial synthesis approaches (Jung, 1994; Haynes, 2006; Kim and Sasaki, 2006; Li et al., 2006; Wang et al., 2014; Corsello and Garg, 2015; Vil et al., 2017), here we shall only focus on reported industrial-scale partial synthesis routes or syntheses conceivably amenable to industrial scale-up that represent or could become commercially viable routes to artemisinin and by extension, the various derivatives used in ACTs. All semi-syntheses involve the steps shown in **Figure 2**, differing only in the reagents used to accomplish each step.

The first partial synthesis (Roth and Acton, 1989) started with a NaBH_4_/NiCl_2_.6H_2_0 (“nickel boride”) reduction of the unsaturated carboxylic acid group of artemisinic acid, a relatively abundant *A. annua* natural product. This reduction afforded an 85:15 (*R*:*S*) mixture of 11,13-dihydroartemisinic acid isomers. 11-(*R*)-Dihydroartemisinic acid has also been reported as present (Wallaart et al., 1999) or not (Haynes and Vonwiller, 1991) in *A. annua*. This apparent contradiction has been explained by either differences in the plant cultivars analyzed or the inherent instability of the molecule, which reportedly quickly vanishes from the leaves after harvest (Kim and Kim, 1992). This would be consistent with a spontaneous transformation of 11-(*R*)-dihydroartemisinic acid into artemisinin on exposure to air and light, conditions likely found in nature (Czechowski et al., 2016) and mimicked by exposing the intermediate to photooxidation, followed by air oxidation in petroleum ether at room temperature for 4 days to give a 17% total yield of artemisinin (Roth and Acton, 1989).

Almost all other subsequent semi-syntheses have followed this route, with variations of the reagents and reaction conditions used in the various steps with the aim of improving the overall reaction yield. These include: (1) the use of asymmetric catalytic hydrogenation in the production of dihydroartemisinic acid aimed at improving the (*R:S*) ratio, as only the *R* isomer correctly forms artemisinin, while the “wrong” (*S*)-isomer undergoes an identical sequence of intermediate steps to give the undesired 9-(*S*)-isomer of the final target; (2) protection of the carboxylic acid function, typically as a simple ester. The advantages of esterification enabled a 23% yield of artemisinin using the methyl ester in place of the acid (Acton and Roth, 1992). Presumably the protection blocks the well-known oxidative lactonization of dihydroartemisinic acid to give dihydro-*epi*-deoxyarteannuin B [the latter is an advanced intermediate in an alternative semi-synthesis of artemisinin (Nowak and Lansbury, 1998), but in the sequence of **Figure 2** it is an unproductive byproduct]; (3) the use of pure oxygen instead of air in the final step, along with the addition of diverse catalysts, such as acid (Kim and Kim, 1992) or copper ion (Kim and Sasaki, 2006).

The first synthesis amenable to scale-up was developed by Amyris chemists in the context of the semi-synthetic artemisinin project described above. As the main details have been reported elsewhere (Paddon et al., 2013) only the highlights are summarized here: (1) the use of chlorotris (triphenylphosphonium) rhodium(I) (“Wilkinson’s catalyst”) in an asymmetric catalytic hydrogenation of artemisinic acid to afford a 90:10 ratio of (*R*) and (*S*)-dihydroartemisinic acid; (2) on the assumption that large-scale photochemistry would add significant capital costs to the project, the dye sensitized photogeneration of singlet oxygen was replaced by a chemical generation of this reactive species based on the group VI metal salt-induced disproportionation of concentrated H_2_O_2_ (Boehme and Brauer, 1992; Nardello et al., 2002); (3) for safety reasons the oxygen used in the last step was replaced by air, and (4) benzenesulfonic acid/Cu(II) Dowex resin was used as catalyst replacing the expensive copper triflate used in other syntheses. This 4-step synthesis gave the desired target in 40% overall yield, an improvement over the typical <30% overall yields previously reported in the literature.

After the technology transferred from Amyris to Sanofi, extensive work was undertaken between 2008 and 2013 to “industrialize” this process. Despite a series of notable improvements to the Amyris route that provided a safe and scalable process that even slightly improved upon the Amyris bench scale yields at pilot scale, it became apparent that this route had reached its performance limits and would not be cost-effective enough for commercial production. This led Sanofi chemists to reconsider the original photochemical approach. As the details of this work have been published (Turconi et al., 2014), including the optimization of the key photochemical generation of singlet oxygen (Burgard et al., 2016), they are not repeated here. The resulting semi-batch tetraphenylporphyrin dye sensitized photochemical process in a custom-designed photochemical reactor is currently being used to manufacture up to 60 MT/year of the target molecule in about 55% overall yield from artemisinic acid produced by fermentation of engineered *S. cerevisiae* strains in what is the only current industrial-scale semi-synthesis of artemisinin.

While as yet unproven as commercially viable syntheses of artemisinin, there have been developments that point the way to potential additional improvements that could break the existing cost/yield barrier. For example, although not part of the current manufacturing process, Sanofi chemists also developed an alternative high-yielding diimide reduction of artemisinic acid to dihydroartemisinic acid that does not require catalytic hydrogenation with a chiral catalyst, yet still provides excellent chemical yields (>90% including all crystallization, isolation, and drying steps), in addition to high diastereoselectivities (≥97:3). Details of the pilot-scale optimization of this process have been published (Feth et al., 2013). In another area, the continuous-flow photochemical synthesis of artemisinin from dihydroartemisinic acid has been reported (Levesque and Seeberger, 2012; Kopetzki et al., 2013). Although to our knowledge this approach has not been scaled up, recent data (Porta et al., 2016) suggest that once the problems of large-scale generation of singlet oxygen in a flow system are overcome (Loponov et al., 2014), this could conceivably become the basis of a new industrial route. Meanwhile, other authors have followed up on the original Amyris “non-photochemical” route and claim similar yields of around 40% using the same molybdate catalyst/H_2_O_2_ route to generate singlet oxygen, but with a simplified and perhaps easier to scale overall process (Chen et al., 2013). Finally, very recently a route that differs in part from the previous approaches has been demonstrated. Chemists at IPCA have reported a novel large-scale synthesis of artemisinin from amorphadiene (Singh et al., 2017). The key step in this route is the functionalization of amorphadiene using simple and cheap chemistry to directly afford (*R*)-dihydroartemisinic acid (i.e., avoiding the need for a stereoselective reduction of artemisinic acid). The reported ca. 60% yield of pure artemisinin from dihydroartemisinic acid obtained also using an improved molybdate/peroxide route suggests that further yield breakthroughs may indeed be possible through additional process optimization of the key steps, namely the singlet oxygen generation and the Hoch cleavage and subsequent rearrangements.

## Conclusion and Outlook

Malaria remains a major disease in the developing world, killing approximately 1500 people per day, the majority being children in sub-Saharan Africa (White et al., 2014). Artemisinin derivatives, administered as ACTs, remain the most effective antimalarial medications. The plant-derived supply of artemisinin has become more plentiful since initiation of the semi-synthetic artemisinin project, but is still subject to the vagaries of weather and agricultural economics; a second-source of artemisinin supply as described here is still required to stabilize the supply chain of this critically important drug. Industrial production of semi-synthetic artemisinin began in 2013, but technological advances described above in both biology and chemistry have opened opportunities to improve the process and decrease the cost of semi-synthetic artemisinin production. Developing these technologies could further safeguard the supply of artemisinin for those who need it most in the developing world.

## Author Contributions

All authors wrote different sections of the mini-review, and the completed manuscript was assembled and edited by the corresponding author (CP). All authors approved the submitted manuscript.

## Conflict of Interest Statement

All authors have shares or stock options in Amyris, Inc.
